# Bypassing or successful referral? A population-based study of reasons why women travel far for childbirth in Eastern Uganda

**DOI:** 10.1186/s12884-020-03194-2

**Published:** 2020-08-27

**Authors:** Paul Mubiri, Darious Kajjo, Monica Okuga, Tanya Marchant, Stefan Peterson, Peter Waiswa, Claudia Hanson

**Affiliations:** 1 Makerere University College of Health Sciences and School of Public Health, Kampala, Uganda; 2 Department of Disease Control, London School of Hygiene & Tropical Medicine, London, UK; 3 Department of Women and Children’s Health, Uppsala University, Uppsala, Sweden; 4 Department of Public Health Sciences – Global Health, Karolinska Institutet, Stockholm, Sweden

**Keywords:** Bypassing, Childbirth, Pregnancy

## Abstract

**Background:**

Delivery in a facility with a skilled health provider is considered the most important intervention to reduce maternal and early newborn deaths. Providing care close to people’s homes is an important strategy to facilitate equitable access, but many women are known to bypass the closest delivery facility for a higher level one. The aim of this study was to investigate to what extent mothers in rural Uganda bypassed their nearest facility for childbirth care and the determinants for their choice.

**Methods:**

The study used data collected as part of the Expanded Quality Management Using Information power (EQUIP) study in the Mayuge District of Eastern Uganda between 2011 and 2014. In this study, bypassing was defined as delivering in a health facility that was not the nearest childbirth facility to the mother’s home. Multilevel logistic regression was used to model the relationship between bypassing the nearest health facility for childbirth and the different independent factors.

**Results:**

Of all women delivering in a health facility, 45% (499/1115) did not deliver in the nearest facility regardless of the level of care. Further, after excluding women who delivered in health centre II (which is not formally equipped to provide childbirth care) and excluding those who were referred or had a caesarean section (because their reasons for bypassing may be different), 29% (204/717) of women bypassed their nearest facility to give birth in another facility, 50% going to the only hospital of the district. The odds of bypassing increased if a mother belonged to highest wealth quintile compared to the lowest quintile (AOR 2.24, 95% CI: 1.12–4.46) and decreased with increase of readiness of score of the nearest facility for childbirth (AOR = 0.84, 95% CI: 0.69–0.99).

**Conclusions:**

The extent of bypassing the nearest childbirth facility in this rural Ugandan setting was 29%, and was associated primarily with the readiness of the nearest facility to provide care as well as the wealth of the household. These results suggest inequalities in bypassing for better quality care that have important implications for improving Uganda’s maternal and newborn health outcomes.

## Background

Globally, an estimated 2.6 million infants die each year during the first 28 days of life [1], including one million babies who die during the day of birth [2]. Seventy-five per cent of deaths of children who die during their first day of life occur in sub-Saharan Africa [2]. An additional estimated 2.6 million third trimester stillbirths occurred globally in 2015 [3]. Of the 303,000 maternal deaths that occur worldwide annually [4], roughly two-thirds occur in sub-Saharan Africa.

Delivery in a facility with a skilled health provider is considered the most important intervention to reduce maternal and perinatal mortality [5], and improving the quality of obstetric care at the closest health care facility to mothers is considered as crucial to reduce maternal and neonatal deaths [6–8]. While the proportion of women giving birth in a facility has increased even in poor and remote settings, large inequities persist [9]. Distance from a mother’s residence to a health care facility has been identified as a main factor hindering access [10, 11]. However, on the other side, “bypassing” of health facilities i.e. delivery in a facility that is not the closest one to a mother’s home has been described in several studies [12–17]. While giving birth in a higher-level facility might provide women with better care and might be safer for the mother and her newborn, overmedicalisation in such facilities has been described and costs maybe higher for both i) the health care system as well as for i) women and their families[18, 19].

Re-evaluating these positive and negative effects has been done within the Lancet Quality of Care Commission which calls to centralise childbirth care to higher level health facilities [20]. The call is supported by several studies describing bypassing: Studies from Tanzania reported that 40–75% of women who delivered in a facility did not deliver in the nearest facility [14, 16, 17]. Studies conducted in Asia have reported the extent of bypassing to be between 37% in Gujarat, India [13] to 70% in Nepal [15]. Reasons for high rates of bypassing put forward are supply side aspects, including a lack of equipment or drugs, skilled health workers, poor physical infrastructure, and lack of facilities to perform operations [12]. Some studies have concluded that perceived quality of care may be the main driver of bypassing [12, 16] while others attribute bypassing to the limited availability of Emergency Obstetric and Neonatal Care services at the nearest health facility in many areas [13, 14]. A major limitation of these studies was that they do not exclude high risk mothers who were referred to higher level facilities for obstetrical care or delivery by caesarean section and did not make a personal decision to bypass the nearest health care facility.

This study investigated bypassing for facility delivery in Uganda, a country where maternal mortality remains unacceptably high at 336 deaths per 100,000 live births. Neonatal mortality has also remained high and stagnated at 27 deaths per 1,000 live births for over a decade despite increases in the proportion of mothers who now deliver in health facilities (73%) and under the care of skilled obstetrical providers (74%) [21]. Rates of stillbirth are estimated to be 1% in Uganda [22]; and a large number of stillbirths are attributable to complications during childbirth [23]. Strategies hitherto have focused on bringing improved maternal health services to designated health facilities close to women in order to reduce travel distances and associated costs to mothers as well as to reduce critical delays in seeking antenatal and birthing care [24].

The health system in Uganda includes public and private sectors. The public health system consists of district health system (health centre (HC) type II, III, and IV, general hospitals), regional and national referral hospitals. National policy stipulates that delivery care should be offered at HC III, IV and hospitals, which should be equipped to provide at least basic emergency obstetric care as well as antenatal and post-natal care. Each health centre is supposed to link to a referring facility - either a HC IV or general hospital that provides comprehensive emergency obstetric care. The private sector includes private-not-for-profit organizations and private health practitioners where services are provided on a fee-for-service basis [25]. Services by private-not-for-profit entities are co-funded by the government through public-private partnership agreements. Although 72% of households in Uganda live within five kilometres from a public or private-not-for-profit health facility, many of these facilities are not mandated to offer childbirth services or services are constraint by insufficient staff and working equipment as well as stockouts of drugs and other health supplies [6, 7, 26]. This may contribute to women bypassing the closest designated delivery facility to delivery in a more distant facility. However, the magnitude, and reasons, for such bypassing rather due to a personal choice than referral is not known.

In view of the difficult choices governments face to balance access to high quality delivery care for poor rural families and limited human resources, equipment and supplies, we aimed to investigate to which extent mothers where no major clinical reason was apparent bypassed their nearest public facility for childbirth care and the determinants for their choice.

## Methods

### Study design and setting

This study used data collected as part of the Expanded Quality Management Using Information power (EQUIP) study in the Mayuge District of eastern Uganda between 2011 and 2014 [27–29]. Mayuge is a rural district on the shore of Lake Victoria about 160 km northeast of Kampala. The district has 26 level II, nine level III and two level four health centres and one private-not-for-profit missionary hospital. The level II HC provide basic outpatient care and are not mandated to provide delivery care; still they provided care to mothers who arrived late in labour. HC III are staffed by a senior clinical officer and three nurse midwives. They offer antenatal care, basic emergency obstetric care and postpartum services for low-risk pregnancies [6, 26]. HC IV offer all services provided at HC III in addition to operative care and laboratory services for low and high-risk pregnancies. Hospitals provide comprehensive emergency obstetric and newborn care services, blood transfusion, and laboratory services. Maternal delivery services are free except for the missionary hospital.

Our study was done using data collected during a trial conducted in Mayuge district evaluating a quality improvement approach. Data was collected via continuous household and health facility surveys [27–29].

## Data

A modular cross-sectional survey of heads of households and a module for women aged between 15 and 49 years who had a completed pregnancy in the previous one year were interviewed during six rounds of data collection conducted between 2011 and 2014. Details on the sampling method and organisation of the survey have been published elsewhere [27, 28]. Briefly, questionnaires included sequences of questions from established tools such as the Demographic and Health Survey [21]. Interviews with household heads sought information on socio-demographic and household characteristics including assets and income. A total of 3,199 women with a completed pregnancy during the prior 12 months were interviewed on their health-seeking behaviour, pregnancy history and outcomes, user perceived quality of care, history and events about the last pregnancy not exceeding a year from the day of the interview. Data were collected using Portable Digital Assistants with inbuilt validation systems to minimize errors. A Garmin Global Positioning System GPS 12 (Garmin Ltd, Olathe, Kansas, USA) was used to collect the actual location of the household in the community. The health facility census included all thirty-seven public health facilities and one missionary hospital in Mayuge. The surveys were conducted in six rounds with each round every four months. The surveys collected data on equipment, drugs and vaccines, services available, staffing levels, and record keeping. The GPS instrument was also used to record the location of each health facility in the district. We excluded private facilities for profit in this survey because they performed less than 15 deliveries per month and were not part of the parent EQUIP study.

## Covariates

In this study, the dependent variable was delivering in a health facility public health that was not the nearest childbirth facility to the mother’s home and delivered in childbirth facilities of any type further away than the nearest childbirth facilities.

We measured bypassing using nearest neighbourhood Distance-Bearing extensions Arcview GIS extensions [30] which measures the straight-line distances. We linked GPS coordinates for the household and nearest health facility and calculated (1) the distance from the household and the nearest facility and (2) the distance between the household and the place of birth or health facility were childbirth occurred. If the difference between the two distances was greater than zero, we coded the status as “bypasser” else 0 “non-bypasser”.

**Fig. 1:**
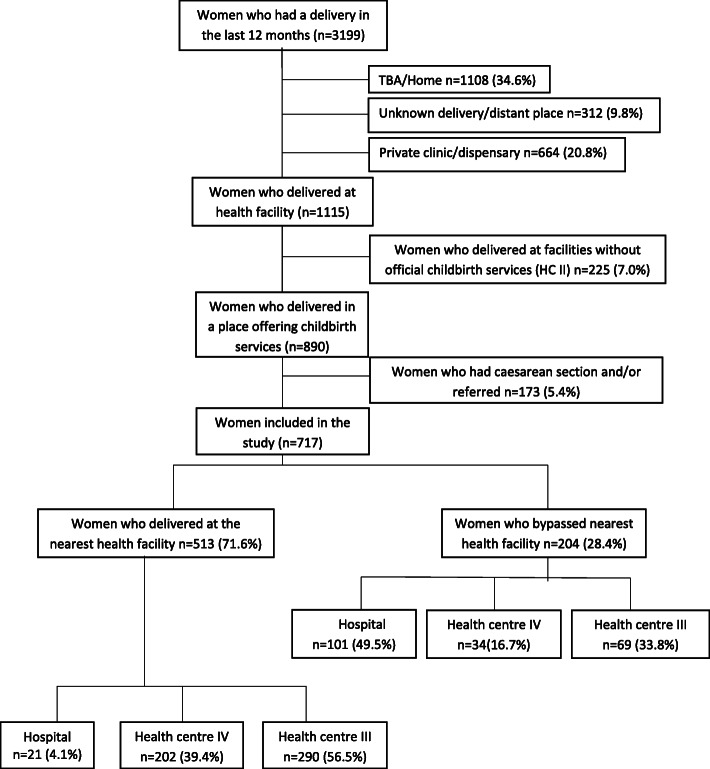
Sample and place of delivery

We explored in our analysis the extent and determinants of bypassing excluding (1) women who did deliver in a HC II (health posts) as these were not officially mandated to offer delivery care (2) and women who were referred away from their nearest facility or had a caesarean section (Fig. 1).

We included 12 independent variables - five socio-demographic variables (age, education, marital status, religion, wealth index), two variables of obstetric risk factors and care characteristics (antenatal care attendance and history of newborn death) and three facility-related factors including healthcare level of nearest health facility, ownership and health facility readiness score which is described below.

A health facility readiness score was generated using 22 items from the health facility survey informed by essential aspects of quality care [29, 31] and a study of community perceptions and preferences with regards to quality of care [32]. Items included the availability of essential drugs (oxytocin and corticosteroids), the presence of water and electricity, referral services, essential equipment, presence of skilled health workers (clinicians, nurses and midwives) and preventive services (malaria, HIV and anaemia). The items included in the readiness score were tested for reliability (α = 0.81). Principal component analysis was used to generate the readiness score. The average score from six repeated health facility censuses were used in the analysis.

A relative wealth index was constructed using principal component analysis from a set of fourteen questions relating to household assets such as the type of toilet facilities, type of fuel for cooking, the source of drinking water, electricity and water. The index was divided into quintiles [33].

### Statistical analysis

Data were analysed using STATA version 14 (Stata Corp, College Station, TX, 2015). Univariate, Bivariate and multilevel model were used to analyse the data. Descriptive statistics included frequencies (proportions) for categorical variables and means (standard deviations-SD) for continuous variables. Chi-square test statistics were used to compare differences between bypassing and categorical variables. A multilevel logistic regression was used to model the relationship between bypassing the nearest health facility for childbirth excluding women who had a caesarean section and/or were referred and the different independent factors to account for clustering nature of our data, and to identify the relative contribution of the facility characteristics to the bypassing phenomenon [34]. Model fitting was carried out in three steps. 1) In Step 1 (Model 1A) only a random intercept was modelled. In Step 2 (Model 1B), individual mother -level variables that had at *p*-value < 0.20) at bivariate analysis or known to be associated with bypassing from literature were added to the intercepts model (age, antenatal care attendance, wealth quintiles). In the final step (Model 1C), facility level variables (ownership and readiness score) were added to Model 1B. Only the facility level variables that had a *p*-value < 0.05 in the bivariate analysis were included in level two of the model. All potential factors were tested for collinearity by examining a correlation matrix and those found to be related one were dropped. In this study, education and wealth were highly correlated, and ownership and type were highly correlated with the readiness score. The final models included only factors predictive of bypassing practice.

## Results

**Fig. 2:**
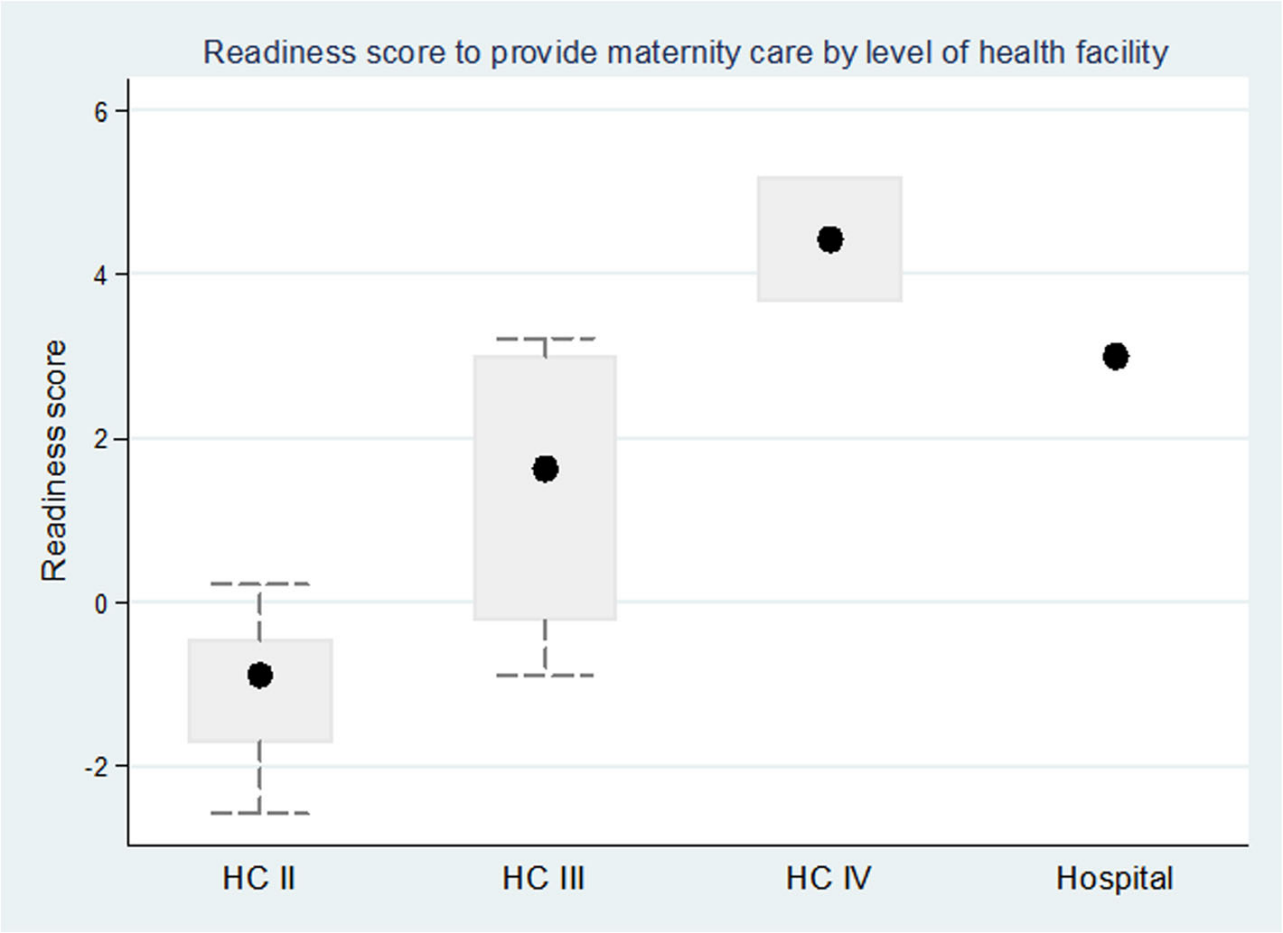
Readiness score by level of health facility

A total of 38 public health facilities had provided delivery care for women in the study area – twenty-six Level II, nine Level III, two Level IV health centres and one hospital. The mean readiness score was 3.01 for the hospital, 4.43 (SD = 1.04) for health centre IV’s, 1.42 (SD = 1.61) for health centre III and − 1.08 (SD = 0.85) for health centre II (Fig. 2). The readiness score differed by level of health facility with the highest score at HC IV (F = 27.15, *p* < 0.001).

Our sample included 3,199 women aged between 15 and 49 years who had a completed pregnancy in the 12 months prior to the survey (Fig. 1), of whom 35% (1,115) delivered is a health facility. Of these, 1,108 (35%) women delivered at home or with a traditional birth attendant (TBA), 664 (21%) delivered at private clinics and 312 (10%) were not able to report on a delivery place; therefore, the sample included 1,115 women who had delivered in a public health facility.

**Fig. 3:**
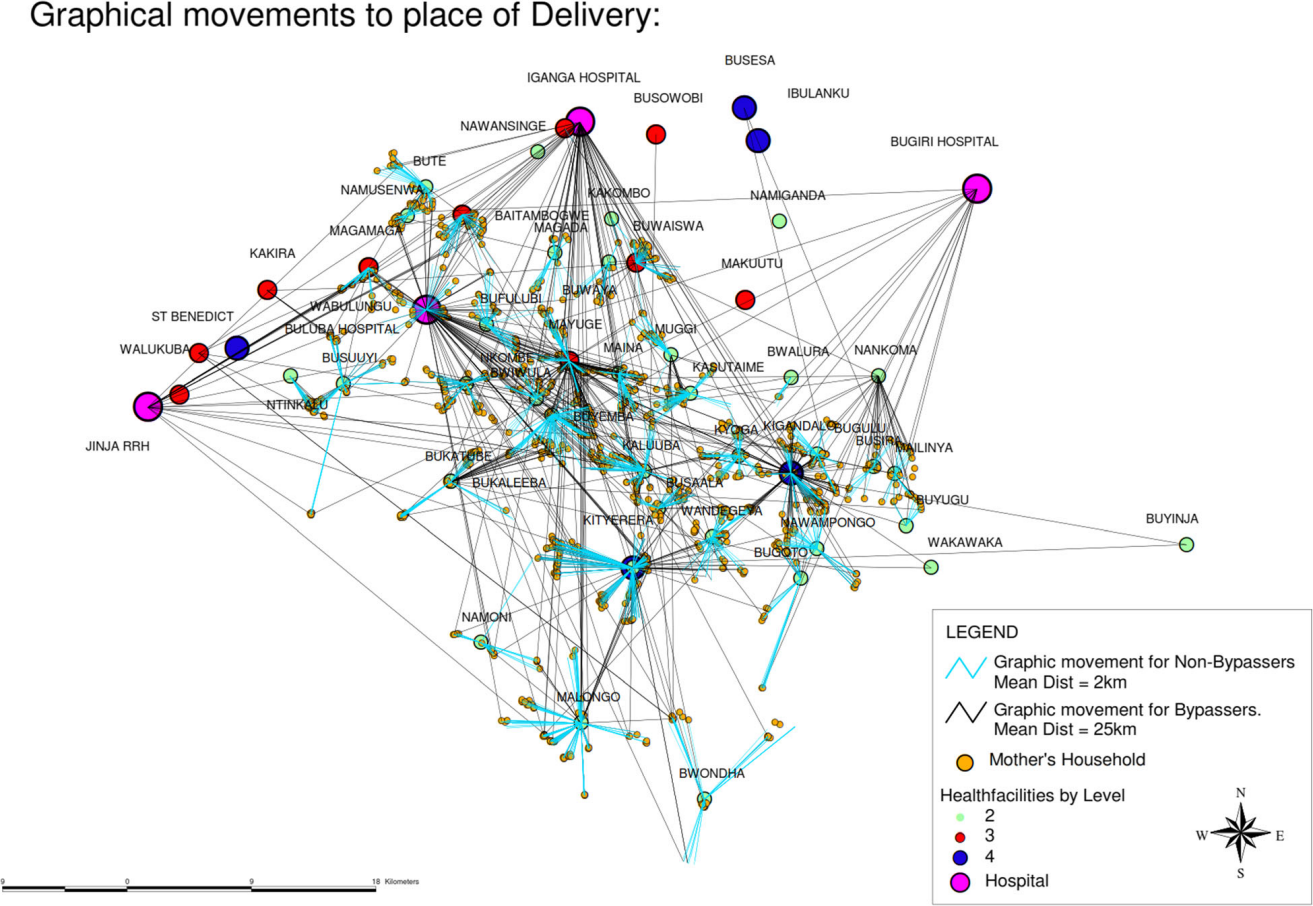
Map showing movements of women in this study graphically

The estimated prevalence of bypassing differed according to the definition applied, as illustrated in Table 1. Of all women delivering in public health facility, 45% (499/1115) did not deliver in the nearest facility regardless of the level of care available there. However, after excluding women who delivered in public facilities not offically designated to provide childbirth services (HCII, *n* = 225) this percentage decreased to 34% (306/890). Further, excluding those who were referred or had a caesarean section, 29% (204/717) of women who delivered in an official public health facility bypassed their nearest facility. Of the 204 women that bypassed the nearest health facility, 50% (101) delivered in a hospital, 17% (34) in a HC IV and 34% (69) in a HC III (Figs. 1 and 3).

**Table 1:** Extent of bypassing the nearest health facility for delivery exploring different definitions for bypassing

Bypassing definition	1) Conventional bypassing Mothers who did not deliver at the nearest facilities regardless of level of care (including Health Center II)	2) Bypassing: Mothers who did not deliver in the nearest facilities regardless of level of care (excluding Health Center II)	3) Bypassing:
			4) Mothers who did not deliver at the nearest facilities regardless of level of care (excluding Health Center II and/or women who had a caesarean section and or were referred)
Numerator	499	306	204
Denominator	1115	890	717
Bypassing rate	44.7%	34.4%	28.5%

The mean age of the 717 women in this study was 27 years. Sixty-one percent had no formal education and 53% were married. Around half had attended four or more antenatal care visits (49%). Slightly more than 1% reported that they had given birth to a live child in the previous one year who had subsequently died (Table 2). Antenatal care attendance and the level of care as assessed by the readiness score at the nearest health facility were associated with bypassing (*P* < 0.05).

**Table 2:** Characteristics of women in the study area stratified by bypassing category excluding women who were referred or had a caesarean section (*n* = 717)

Characteristic	Overall (*n* = 717)	Bypassing status			
		Bypasser (*n* = 204)	Non-bypasser (*n* = 513)	Chi-square value	*P*-value
Age (years)				2.41	0.492
< 20	16.3	19.6	15.0		
20–24	23.6	23.0	23.8		
25–34	44.9	42.2	46.0		
35+	15.2	15.2	15.2		
Education				1.39	0.499
None	61.1	57.8	62.4		
Primary	34.4	37.7	33.1		
Secondary+	4.5	4.4	4.5		
Married	52.6	52.9	52.4	0.14	0.903
Religion				2.41	0.121
Christian	59.4	54.9	61.2		
Muslim or other	40.6	45.1	38.8		
Wealth index				6.74	0.150
Quintile 1 (poorest)	10.3	7.3	11.5		
Quintile 2	14.5	15.2	14.2		
Quintile 3	19.4	19.6	19.3		
Quintile 4	25.5	22.1	26.9		
Quintile 5 (richest)	30.3	35.8	28.1		
ANC attendance				4.63	0.031**
< 4 times	51.5	45.1	54.0		
4 + times	48.5	54.9	46.0		
History of neonatal death	1.3	2.5	0.8	3.29	0.128
Level of the nearest health facility				17.25	0.001***
HC III	45.1	33.8	56.5		
HC IV	28.1	16.7	39.4		
Hospital	26.8	49.5	4.1		
Ownership of the nearest HF				3.75	0.053*
Government	87.2	83.3	88.7		
Private	12.8	16.7	11.3		
Mean Readiness score of the nearest health facility (SD)^+^	2.6 (1.9)	2.1 (1.8)	2.8 (1.9)	4.82	0.001**

***+ Z – test statistics (Wilcoxon rank test)***

The overall odds of bypassing the closet childbirth facility after excluding women who delivered in facilities not officially designated to provide childbirth services and/or women who had a caesarean section or were referred in this study was 0.55 (Model 1A random intercept) (Table 3). In model 1B, individual-level covariates that showed an association with bypassing in the bivariate analysis were included. However, only being a member of a household from the highest household income quintile remained associated with bypassing (adjusted OR 2.24, 95% CI 1.12–4.46). The odds of a mother bypassing the nearest facility doubled if the mother belonged to highest wealth quintile compared to the lowest quintile (*AOR* = 2.24, 95% CI: 1.12–4.46). Furthermore, women who attended 4 or more antenatal visits were 36% more likely to bypass the nearest health facility for delivery (*OR* = 1.36, 95 CI: 0.96–1.93). In model 1C, we adjusted for individual level variables and included the health facility readiness score variable. With increasing facility readiness score, the odds of bypassing the nearest health facility decreased by 16% (*AOR* = 0.84, 95% CI: 0.69–0.99). Being part of a household belonging to the least poor wealth quintile continued to impact bypassing behaviour compared to the lowest wealth quintile. The odds of bypassing were almost three times higher in this group compared to mothers from the lowest wealth quintile.

**Table 3:** Multilevel logistic regression of the odds of bypassing the nearest health facility for childbirth (*n *= 717)

Covariate	Model 1A		Model 1B		Model 1C	
	**Null model (*n* = 717)**		**Individual level covariates**		Individual level + nearest health facility covariates (*n* = 688)	
	**OR**	**95% CI**	**OR**	**95% CI**	**OR**	95% CI
*Level 1*						
Age category						
< 20			1		1	
20–24			0.67	0.38–1.15	0.66	0.38–1.15
25–34			0.63	0.38–1.03*	0.61	0.37–1.01*
35+			0.69	0.37–1.28	0.63	0.33–1.18
Wealth quintiles						
1 (poorest)			1		1	
2			1.69	0.79–3.59	2.00	0.91–4.44*
3			1.93	0.94–3.97*	2.01	0.95–4.37*
4			1.40	0.68–2.85	1.68	0.79–3.56
5 (richest)			2.24	1.12–4.46**	2.73	1.32–5.65**
Antenatal attendance						
< 4 times			1		1	
4 + times			1.36	0.96–1.93*	1.34	0.94–1.91
History of neonatal death						
No			1		1	
Yes			2.42	0.59–9.78	2.51	0.63–10.09
*Level 2*						
Readiness for nearest health facility					0.84	0.69–0.99**
Constant	0.55	0.33–0.91	0.38	0.16–0.89	0.39	0.16–0.95
Model statistics	Var (_cons) = 0.69		Var (_cons) = 0.72 Wald chi2 (6) = 14.62 Prob > chi2 = 0.102		Var (_cons) = 0.23 Wald chi2 (7) = 19.09 Prob > chi2 = 0.039	

***p < 0.001, **p < 0.05, *p < 0.1

## Discussion

In our study conducted in a rural district in eastern Uganda, when excluding mothers who delivered at home or private clinics or were referred to a specialized facility or had a caesarean section, we observed that 29% of women bypassed their nearest public health facility, typically a HC III, for delivery care at a higher level HC VI or the hospital. Household wealth status and the quality of obstetrical and pediatric care available at the nearest facility were the major predictors of bypassing in this study.

Our estimate of bypassing behaviour was lower than that reported by other studies. Previous studies from Tanzania, Nepal and India reported the prevalence of bypassing the nearest health facility of between 40% − 70% [12–14, 16, 17, 35]. The lower rate which we report could be partly have been driven by our exclusion of women who had a Caesarean section and mothers who self-reported that they were referred to specialized centres for delivery. Other studies reported rates including those women who were referred [13, 14, 16, 17, 35]. Even if we excluded stated referrals, we cannot exclude the possibility that some women sought care at higher level facilities because they felt at risk for complications but were not referred by health care workers at the facility where they received antenatal care. Thus, we might have overestimated bypassing rates. Several studies on bypassing have reported that primigravidae are more likely to seek care at higher level facilities and hospitals [14–16]. Primigravidae have a higher risk for obstetrical complications so their decision to bypass may be quite rational.

While we report that 29% of women bypassed the nearest facility, 35% delivered at home and 7% in a HC II near home indicating that limited access to obstetrical services is still a major issue in rural Uganda, as also reported in the latest demographic health survey [21].

We found that household wealth was predictive of bypassing. This is similar to other studies from Tanzania and Nepal [12, 15, 16]. Women from wealthier households may prefer to seek care at high-level facilities like hospitals because they can afford these facilities and the associated costs. In India, households of higher wealth quintiles also cited quality concerns as basis for their bypassing [20].

In the bivariate analysis we observed an association between completing four or more antenatal care visits and seeking care at higher level facilities. Similar findings were also reported from Nepal but not from Tanzania [15, 17]. Women who attended more than four antenatal care visits may have received more information on the importance of quality delivery care. However, attending more antenatal care visits may also a marker of women who have a higher perceived risk for complications during pregnancy and delivery leading to seeking delivery care at a higher-level facility.

Our results showed that with increased readiness score levels at the nearest health facility, the odds of bypassing a facility for childbirth decreased. Thus, women seem to understand and value the included quality aspects. This is similar to previous studies that have used Emergency Obstetric and Neonatal Care signal functions as a measure to assess health facility preparedness to provide obstetric health services [13, 14, 36]. The large percentage of mothers we observed bypassing and delivering in hospitals (62%), may highlight perceived deficiencies in quality of obstetric care at the lower level health facilities in Uganda. However, an alternative explanation may be risk factors which led mothers to seek care at the hospitals although they were not formally referred. Other studies have used the designated level of care or level of the health facility; however, our analysis indicates that a high degree of variability in this score even among facilities within the same level.

Noting the recommendation in the recent Lancet Quality of Care Commission for deliveries to take place in hospitals and specialized health centres [20], we see this already taking place as many Ugandan women bypass their closest delivery facility, even without a formal policy in place. However, despite the fact that we indicated a high rate of bypassing,in this setting the majority of women continue to deliver at lower levels of the health system with only 11% (358 of the 3,199) of total deliveries taking place in HC IVs and the District Hospital. A change in policy for all deliveries to happen at these higher level facilities would require investment commensurate with a nine-fold increase now 11% of deliveries to 100%.

Our study has several limitations: First, we did not include several important factors such as parity, gravidity, and complications from previous delivery that have been reported to influence the place of childbirth but was not available from the parent study. Second, we defined the quality of the health facility using a latent score including 22 input variables. Data on important process components such as responsiveness to needs and ability to respond to complications such as caesarean section services or other Emergency Obstetric Care functions were not available. Still, we believe that this score is a better indication of quality of care than the emergency obstetric and neonatal care signal functions, as the quality of care at facilities with a low case load like health centres cannot be accurately assessed using interventions carried out for relatively rare complications. The only hospital in the study area did not score better than other lower level facilities because it did not have motorized referral services or supplemental oxygen or the capacity to perform vacuum extractions. Third, we also excluded women who delivered a baby in the year prior to the survey who delivered at a facility that is does not officially offer childbirth care. The fact that many mothers choose to deliver at such facilities could indicate a preference for proximity, and/or a lack of understanding of requirements for safe delivery care. Fourth, we defined our dependent variable using straight line distances that may underestimate the actual travel distance between the household and the nearest birth facility. Fifth, our analysis is based on cross-sectional design that cannot prove the predictors represented causal relations. Lastly, during our parent study, another quality improvement project was being conducted in the same area which aimed to improve the quality of care in primary and referral facilities [27–29]. We cannot exclude that this affected the women in our study’s preferences on where to deliver, but as referral aspects were not part of the study, we do not believe that the intervention had a major impact on bypassing.

## Conclusions

After adjustment for referrals and women seeking caesarean sections, one-third of women in this rural Ugandan setting did not use the nearest facility for childbirth care. This population-based study provides insights into women’s preferences on where to deliver and how the quality of obstetric care at the nearest facility influence that choice notwithstanding the complexities and other factors that could influence the choice of the facility for delivery. These results suggest inequalities in bypassing for better quality care that have important implications for improving Uganda’s maternal and newborn health outcomes and proposed changes in the public health care system in Uganda to centralise childbirth care at higher level facilities.

## Data Availability

The datasets used and/or analysed during the study are available from the corresponding author on reasonable request.

## Abbreviations

TBA: Traditional Birth Attendant
PNFP: Private-Not-For-Profit
DHS: Demographic Health Survey
GIS: Geographical Information System
HC: Health Centre
EQUIP: Expanded Quality Management Using Information Power
GPS: Global Positioning System
